# CHARACTERIZING HOME AND COMMUNITY-BASED SERVICE ENROLLEES IN MINNESOTA’S MEDICAID WAIVER PROGRAM

**DOI:** 10.1093/geroni/igac059.097

**Published:** 2022-12-20

**Authors:** Justin Blackburn, Sharon Baggett, Ellen Burton, Yonda Snyder

**Affiliations:** IU Richard M. Fairbanks School of Public Health at IUPUI, Indianapolis, Indiana, United States; University of Indianapolis, Indianapolis, Indiana, United States; University of Indianapolis Center for Aging & Community, Indianapolis, Indiana, United States; Sage Squirrel Consulting, LLC, Bloomington, Indiana, United States

## Abstract

Medicaid waivers allow states’ provision of home and community-based services (HCBS), leading to variations in design and delivery. States monitor expenditures, but cannot easily anticipate growth. Understanding the needs of this population can aid in early identification and improve service delivery. We conducted a Partitioning Around Medioids cluster analysis of first-time enrollees in Minnesota’s Elderly Waiver HCBS program during 2019 to identify trajectories of entry and characterize activities of daily living (ADL) and instrumental activities of daily living (IADL) needs at enrollment. Administrative data collected via long term care consultation assessments provided enrollment, ADL/IADL needs, living arrangements, and other clinically relevant information that was linked to other sources including Minnesota health care program enrollment and utilization, Minimum Data Set skilled nursing facility (SNF) assessments, and calls to the Senior LinkAge Line (SLL)—a free long-term care counseling service. Of 5,284 first-time enrollees, most had prior engagement with state programs—nearly two-thirds had called the SLL, 36% had a SNF stay, and 56% had prior Medicaid enrollment. We identified six clusters representing three levels of living arrangements and two levels of need: 1) lived alone, low needs (32%), 2) lived with others, moderate needs (20%), 3) congregate living, moderate needs (14%), 4) congregate living, high needs (14%), 5) lived with others, high needs (11%), and 6) lived alone, high needs (9%). Lacking caregivers and prior Medicaid were possible exacerbating reasons for HCBS enrollment. The magnitude of the differences between clusters highlights the constellation of factors leading to enrollment in HCBS.

